# Prediction of Tetraoxygen Reaction Mechanism with Sulfur Atom on the Singlet Potential Energy Surface

**DOI:** 10.1155/2014/912391

**Published:** 2014-01-23

**Authors:** Ashraf Khademzadeh, Morteza Vahedpour, Fereshte Karami

**Affiliations:** Chemistry Department, University of Zanjan, Zanjan 45371-38791, Iran

## Abstract

The mechanism of S+O_4_ (D_2h_) reaction has been investigated at the B3LYP/6-311+G(3df) and CCSD levels on the singlet potential energy surface. One stable complex has been found for the S+O_4_ (D_2h_) reaction, IN1, on the singlet potential energy surface. For the title reaction, we obtained four kinds of products at the B3LYP level, which have enough thermodynamic stability. The results reveal that the product P3 is spontaneous and exothermic with −188.042 and −179.147 kcal/mol in Gibbs free energy and enthalpy of reaction, respectively. Because P1 adduct is produced after passing two low energy level transition states, kinetically, it is the most favorable adduct in the ^1^S+^1^O_4_ (D_2h_) atmospheric reactions.

## 1. Introduction

The oxygen atom exists naturally in four basic forms including free atomic particle, diatomic oxygen O_2_, ozone O_3_, and tetraoxygen O_4_. Tetraoxygen is typically an unstable, rare, nonmagnetic pale blue gas. Interest in tetraoxygen molecules dates to a 1924 by Lewis [1]; since then, studies of O_4_ known as “oxozone” have enjoyed a long history of investigation [2–10].

Since its discovery, O_4_ has been a subject of interest from both experimental and theoretical point of views. The first experimental study of molecular oxygen by using spectroscopic method came back to Arnold and coworker's research [11]. They suggested the existence of O_4_ molecule as van der Waals complex. In addition, Helm and Walter [12], are observed formation of O_4_ molecules when electron transferred to form of O_4_^+^. The kinetic energy release and the nature of dissociation have been investigated using electronic excited state. The metastable structure of O_4_ molecule has been recently observed in mass spectroscopy experiments by Cacace et al. [13]. Then, Bevsek et al. [14] described 1 : 1 (or 1+1) resonant photoionization spectra of an energetic metastable O_4_ species produced in a dc discharge. In another study, Adamantides et al. defined a long-predicted covalent forms, either cyclic, D_2d_, or pinwheel, D_3h_, structures [15].

In theoretical approach, Adamantides et al. (1980) [15] predicted the existence of metastable covalent O_4_ molecule completely different from the van der Waals structure of O_2_–O_2_ molecule which was experimentally detected. Then, Røeggn and Nilssen (1989) [16] have shown a branch form of O_4_ analogues with the stable isoelectronic molecules NO_3_^−^ and OO_3_. A central atom and three equivalent ligand atoms characterized it, which can exist in metastable D_3h_ form. In 1990, Dunn et al. [6] predicted a cyclostructure for O_4_ molecule with the bond length of 1.437 Å. In addition, dissociation of cyclostructure O_4_ molecule is investigated by Seidl and Schaefer [9] based on new high-energy materials. An ab initio calculation has been done on the ground and low-lying electronic states of O_4_ along the minimum energy to explain the reactivity and electronic state of tetraoxygen. Obtained results provided a solid basis to establish the stability of the O_4_ chemically bound molecule. In addition, surface crossings between singlet and triplet states were found and further characterized by evaluating their spin-orbit coupling matrix elements [7]. Peterka [5] present rotationally resolved photoionization spectra, photoelectron spectra, and ab initio calculations for providing strong evidence for the identity of this species as a novel complex between a ground state molecule and one excited state. In summary, theoretical calculations have predicted the existence of metastable O_4_ molecules with two different shapes: cyclobutane and trigonal planar similar to boron trifluoride. The existence of O_4_ was further confirmed in some other theoretical and experimental research [14, 17–20].

On the other hand, processes such as volcanic eruptions, biogenic activity, and fossil fuel combustion all contribute to the emission of sulfur gases into the atmosphere. Sulfur has long been recognized as an important constituent of atmospheric aerosols and sulfur compounds are major pollutants of the environment [21]. The sixteen molecular species of type S_*n*_O_*m*_ (SO, SO_2_, SO_3_, SO_4_, etc.) have been detected experimentally [22]. Among these species, the sulfur dioxide (SO_2_) molecule plays an important role in the atmospheric formation of sulfuric acid.

Sulfur tetraoxide (SO_4_) as an interesting atmospheric compound is potentially relevant to atmospheric chemistry [23]. McKee [24] considered several structures for the SO_4_ species and predicted that the lowest-energy structure is to have a three-membered SOO ring and C_2v_ symmetry. Schriver et al. [25] also reported IR bands at 1,442 and  1,272 cm^−1^, which they attribute to SO_4_. Kugel and Taube [26] give a discussion of the synthesis and characterization of the monomeric SO_4_. Previously, sulfur tetraoxide has been proposed as intermediate in a variety of sulfur oxide reaction systems, as the active oxidizing species in per-sulfate oxidations [27]. Therefore, the experimental and theoretical studies reveal that the most stable structure for SO_4_ is a three-membered ring (SOO) with two terminal oxygen atoms attached to ring sulfur with C_2v_ symmetry (SO_4_  (C_2v_)).  SO_4_  (C_2v_) molecule has been confirmed in both the experimental and theoretical studies, while SO_4_  (C_3v_) molecule has been only confirmed in the theoretical studies.

To our knowledge, there is not any theoretical or experimental work on the reaction mechanism of S+O_4_ reaction. Hence, in this work the reaction mechanism of sulfur atom reaction with O_4_ molecule on the singlet potential energy surface are investigated. Then, thermodynamic parameters of the reaction are obtained and results the reaction is spontaneous with the releasing of the energy. As well as, the structure of O_4_ molecule and possible products are reported on the singlet potential energy surface.

## 2. Computational Details

All the calculations are performed with the Gaussian 03 system of codes [28]. The geometries of all species involved in the ^1^S+^1^O_4_ reaction are fully optimized at the B3LYP [29] level in conjunction with the 6-311+G (3df) basis set. The 6-311+G (3df) basis set has been used because of the lack of hydrogen atoms in structures. These energies are corrected by zero point energies of B3LYP level. In present study the coupled cluster singles and doubles (CCSD) method has been employed [30, 31]. This method has the advantage of being able to evaluate the correlation surface in a size consistent manner. Hence, the CCSD calculations are carried out to compute accurate relative energies. The counterpoise procedure has been used to correct the interaction energy for basis set superposition error, BSSE, for the structures. So, the obtained energies are corrected by BSSE energies.

At the motioned level of treatment, we computed the vibrational frequencies to determine the nature of the stationary points according to the number of the eigenvalues of the Hessian matrix. The results show that one of reactants (O_4_), products, and intermediates possesses all real frequencies, and any transition state has only one imaginary frequency. The imaginary frequency that characterizes each transition state is included as well. Another reactant, S, has no vibrational frequencies. To verify that the TS structure is the right saddle point connecting the corresponding reactants and products of interest, the intrinsic reaction coordinate, IRC [32], calculations were performed at the B3LYP level. These calculations show that all TS structures of title reactions are valid. At the level of this method, it is possible to generate a wave function in a suitable form to execute a topological analysis of atoms in molecules [33] using the AIM2000 series programs [34]. Moreover, thermodynamic data have been calculated using the statistical mechanics.

## 3. Results and Discussion

**Figure 1:**
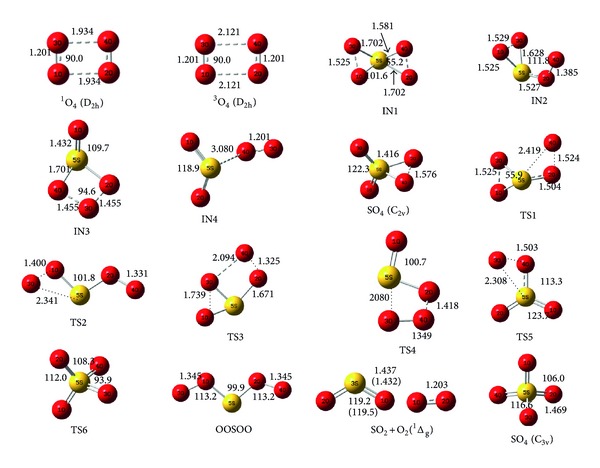
Geometries of reactants, products, intermediates, and transition states optimized on the singlet PES at the B3LYP level (bond distances are in angstrom and angles are in degree). The values in parentheses refer to experimental data [35].

**Figure 2:**
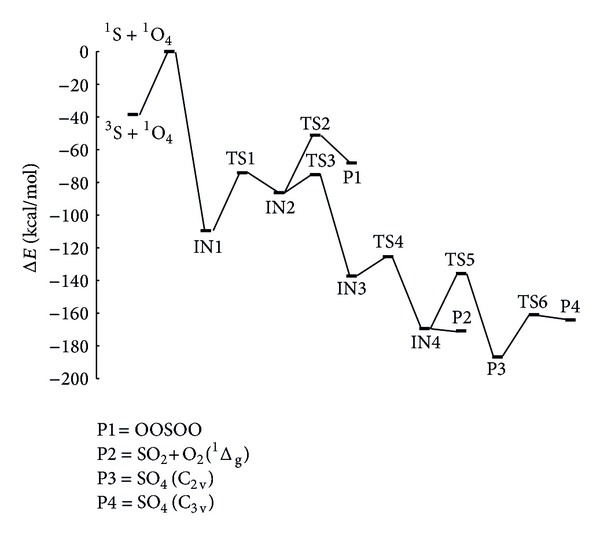
Profile of calculated potential energy surface of ^1^S+^1^O_4_ reaction at B3LYP level of computation.

**Figure 3:**
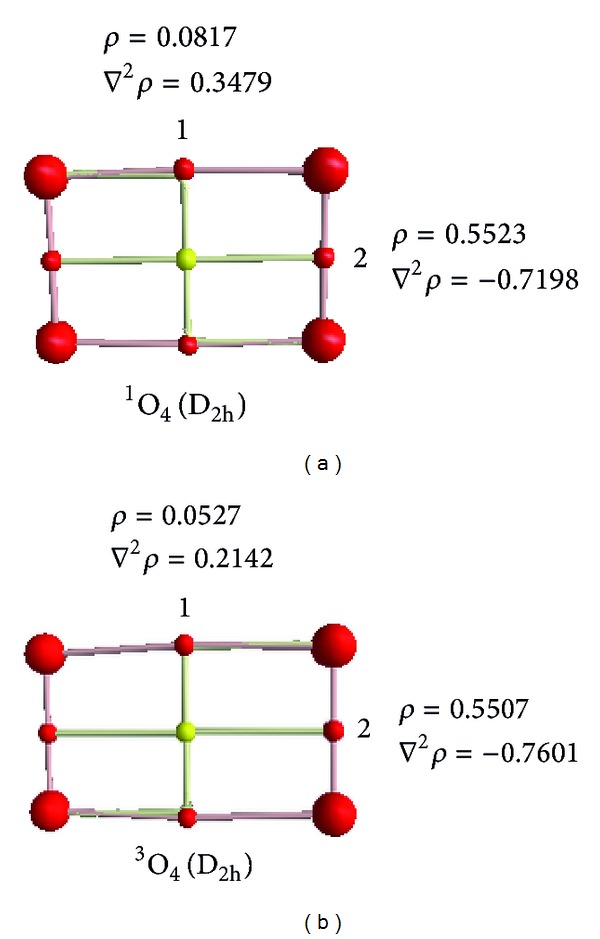
The values of the AIM theory topological parameters for bonds of tetraoxygen on the singlet and triplet PESs.

**Figure 4:**
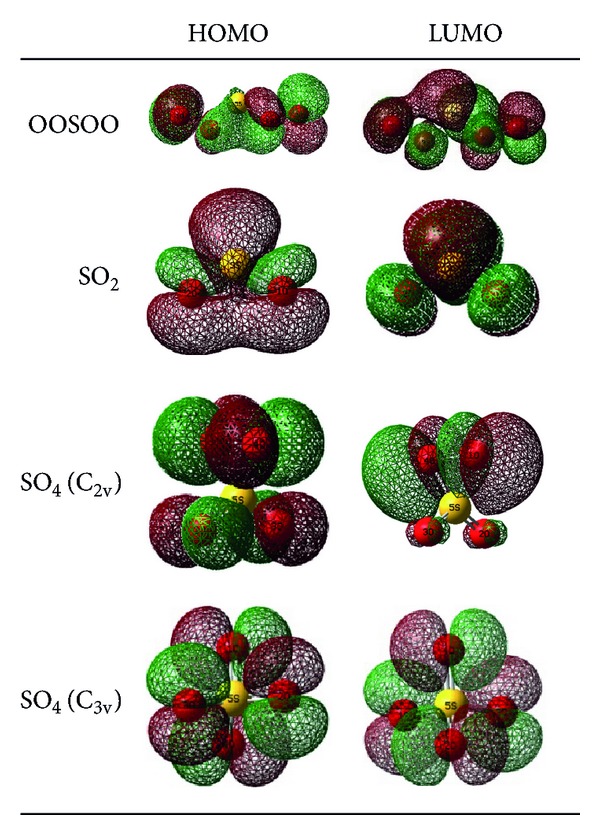
Frontier molecular orbitals of OOSOO, SO_2_, SO_4_ (C_2v_), and SO_4_ (C_3v_) molecules.

The reaction mechanisms of S with O_4_ have been investigated on singlet PES. One stable complex has been found between the reactants. Hence, all of the elementary reactions considered in the title reaction begin with the stable initial complex after passing corresponding transition states to produce final adducts. The intermediates prefix is IN followed by a number to differentiate one from each other. The transition states are designated by the prefix TS and a number. The structures of the reactants, products, intermediates, and transition states involved in the ^1^S+^1^O_4_ reaction are shown in Figure 1. Their Cartesian coordinates are available in the Supplementary Material, available online at http://dx.doi.org/10.1155/2014/912391. By means of the TSs and their connected intermediates (or products), a schematic PES for the reaction is plotted in Figure 2.  Table 1 gives the total energies for the B3LYP and CCSD levels and corresponding relative energies in comparison with the reactants in the singlet state. To simplify our discussion, the energy of reactant R, ^1^S+^1^O_4_, is set to be zero for reference. The AIM theory topological parameters for O_4_ as a reactant on the B3LYP level are exhibited in Figure 3. In addition, the topological characteristics in the bond critical points in the mentioned molecules are tabulated in Table 2. The vibrational frequencies of all species are summarized in Table 3. In addition, the values of the AIM theory topological parameters for selected bonds of products on the B3LYP level are shown in Figure 3. Then, the topological characteristics in the bond critical points, BCPs, in the molecules are tabulated in Table 4. The highest occupied molecular orbital, HOMO, and the lowest unoccupied molecular orbital, LUMO, energies and band gap energy for HOMO to LUMO transition of the selected species for products are shown in Table 5. In addition, frontier molecular orbitals of the same molecules are shown in Figure 4. Finally, the standard thermodynamic functions at room temperature and atmospheric pressure are tabulated in Table 6.

### 3.1. O_**4**_ Molecular Property

Up to date, the experimental and theoretical studies show that O_4_ has different structures with various group points. In addition, its bond strengths and characteristic (van der Waals or covalent bonds) are discussed [7, 12, 16]. In our study, we applied the new structure of neutral tetraoxygen with group point of D_2h_. Of course, in study of Helm and Walter, D_2h_ group point of O_4_ with positive charge has been investigated. O_4_ (D_2d_) has stability energy of −299.0706 au while its structure with the D_3h_ group point shows stability energy of −300.0949 au. In addition, in the mentioned study of Helm and Walter, the bond length of O–O in the O_4_ (D_2d_) is reported 1.433 Å and angle of O1O2O3 atoms is 87.0° while in the O_4_ with D_3h_ point group, the O–O bond length is obtained 1.330 Å and angle of three mentioned atoms is 120.0°. In O_4_^+^ (D_2h_), the bond lengths are 1.160 and 2.396 Å, which shows rectangular shape of molecule [12].

In the O_4_ (D_2h_) on the singlet PES, our results confirmed that the bond lengths are 1.934 and 1.201 Å with rectangular structure of molecule. The stability energy of that is −300.6897 au, which is stable than the other structures with diverse group points on the singlet PES.

The bond formation between the oxygen atoms in the tetraoxygen molecule on the singlet and triplet states is confirmed by atoms in molecules topological analysis of wave function. This analysis reveals the presence of bond critical points located between O_1_–O_2_ atoms in ^1^O_4_ (D_2h_) with density of the wave function: *ρ*(*r*_*bcp*_) = 0.0817 au and the Laplacian of electronic density at critical point, ∇^2^ *ρ*(*r*_*bcp*_) = 0.3479 au. The results confirmed that van der Waals interaction between the O_1_–O_2_ atoms. Although, the AIM analysis reveals covalent bond formation between the O_2_–O_4_ with the Laplacian of electronic charge density of ∇^2^ *ρ*(*r*_*bcp*_) = −0.7198 au. As shown in Figure 3, furthermore, we are analyzed the structure of tetraoxygen on the triplet PES. AIM analyses of ^3^O_4_ (D_2h_) confirm the presence of one BCP located between O_1_–O_2_ with *ρ*(*r*_*bcp*_) = 0.0527 au, and ∇^2^ *ρ*(*r*_*bcp*_) = 0.2142 au. The results display the van der Waals interaction between O_1_–O_2_ atoms. In addition, another BCP is located between O_2_–O_4_ atoms in ^3^O_4_ (D_2h_) molecule with the electronic density *ρ*(*r*_*bcp*_) = 0.5507 au, and the Laplacian of electronic density ∇^2^ *ρ*(*r*_*bcp*_) = −0.7601 au. The AIM analysis shows the covalent bond formation between the O_2_–O_4_ atoms. So, this work suggestion is two van der Waals interaction and two covalent bonds in the structure of O_4_ (D_2h_) molecule on the singlet and triplet states. The relative energies and Laplacian of electronic charge density analysis confirmed that the tetraoxygen molecule on the triplet state with D_2h_ point group is more realable than the other suggested structures.

### 3.2. Initial Interactions of Reactants

Since the ground state of sulfur atom is triplet, the mechanism of S+O_4_ reaction is considered on the triplet PES. In spite of numerous attempts, we could not find any transition state for the reaction on the triplet PES. Only two complexes between the reactants are found on the triplet PES. Therefore, our attempt focused on the singlet PES for finding some intermediate, transition state, products, and suggested mechanism for the reaction pathway at the B3LYP level. Therefore, in the first step, the sulfur atom in triplet state, ^3^S, excited to the singlet state, and the reaction can continue in the singlet state This is explained as follows.

One stable reactant complex, IN1 (O_2_–S–O_2_), has been considered between the reactants on the singlet PES. The intermediate IN1 is formed when the sulfur atom bridged to the oxygen atoms of O_4_ using the formation of four van der Waals bonds and rupture two O_2_–O_2_ van der Waals interactions in O_4_ molecule. In the intermediate IN1, the newly formed S–O bonds are two types. One, bond lengths are 1.525 $\overset{´}{Å}$ and two they are 1.702 $\overset{´}{Å}$ at the B3LYP level with the formation relative energy of −109.524 kcal/mol. No transition states have been found for the formation of IN1 intermediate. Therefore, the results show that the formation of IN1 is a barrier-less and exothermic process. In the complex, four oxygen atoms of O_4_ are associated with sulfur atom and two three-membered rings are formed (see IN1 structure in Figure 1). In the first ring formation the lone pair of the O_2_ and O_4_ atoms of tetraoxygen interacts with the sulfur atom and in the second ring O_1_ and O_3_ atoms associate with the atomic sulfur. Molecular geometry indicates that the IN1 complex has C1 symmetry.

The stable reactant complex formation is confirmed by AIM topological analysis of wave function. Our analysis reveals the presence of bond critical points; one located between two oxygen atoms of O_4_ molecule and sulfur atom with density of wave function, (*ρ*(*r*_*bcp*_) = 0.1698 au and ∇^2^ *ρ*(*r*_*bcp*_) = 0.0245 au) and the other located between two other oxygen and sulfur atoms (*ρ*(*r*_*bcp*_) = 0.2267 au and ∇^2^ *ρ*(*r*_*bcp*_) = 0.0196 au). Therefore, all the O ⋯ S interactions are of the van der Waals types. In addition, the topological analysis of the wave function shows the existence of two bond critical points; one is located between O_1_ and O_3_ (*ρ*(*r*_*bcp*_) = 0.2300 au and ∇^2^ *ρ*(*r*_*bcp*_) = 0.2334 au), and the other is located between O_2_ and O_4_ with the same electronic density and Laplacian. Calculated results allow us to classify the O ⋯ O interactions as being of the van der Waals type. The rings formation between O_2_S_5_O_4_ and O_1_S_5_O_3_ atoms also is lightened using AIM analysis. The topological analysis of the wave functions and their Laplacians are *ρ*(*r*_*bcp*_) = 0.1449 au and ∇^2^ *ρ*(*r*_*bcp*_) = 0.4256 au, respectively.

### 3.3. Formation Pathways of P1 (OOSOO)

There is one possible pathway for production of P1 as follows:

 Path 1: R → IN1 → TS1 → IN2 → TS2 → P1 (OOSOO).

As is shown in path 1, the singlet reactants of S and O_4_ directly transformed to IN1 without entrancing any transition state. Then IN1 transformed to IN2 after passing TS1 with the energy barrier of 35.429 kcal/mol at the B3LYP level. In this step, one O–S van der Waals bond of three-membered ring structure in IN1 molecule ruptures and IN2 appears. IN2 is 86.279 kcal/mol lower than the ^1^S + ^1^O_4_ original reactant. TS1 has one imaginary frequency at 378i cm^−1^ that is vibrated in the reaction coordinate. TS1 has a three-membered ring structure (see Figure 1). The electronic charge density and its Laplacian in ring critical points are 0.1712 and 0.4378 au, respectively. The intermediate IN2 converted into final product after passing TS2 with the energy barrier of 35.155 kcal/mol and imaginary frequency of 163i cm^−1^ in the reaction coordinate. The S–O bond of three-membered ring complex, IN2, is ruptured and converts to the final product P1. The conversion is an endothermic process but the overall reaction from singlet reactants is exothermic with the reaction enthalpy −68.286 kcal/mol and is spontaneous with the Gibbs free energy −62.177 kcal/mol. All steps of reaction as formation of ring structure and its collapse are confirmed by electronic charge density analysis using AIM calculation results. Formation channel of product P1 with two transition states and lower level of energy barriers is favorable process from kinetic point of view.

### 3.4. Formation Pathways of P2 (O_**2**_ (^**1**^Δ_**g**_) + SO_**2**_)

There is one possible pathway for production of P2 as follows:

 Path 2: R → IN1 → TS1 → IN2 → TS3 → IN3 → TS4 → IN4 → P2 (^1^O_2_ (^1^Δ_g_) + SO_2_).

As shown in Figure 2, formation of the intermediate IN2 is similar to path P1; then, it transforms into IN3 using TS3 with energy barrier of 11.048 kcal/mol and imaginary frequency of 591i cm^−1^. The O_2_–O_4_ bond in ring structure of TS3 is 51% longer in comparison with the corresponding bond in IN2. Four-membered ring complex, IN3, (see Figure 1) undergoes elongation of O_3_–S bond and collapse of four-membered ring structure turn to IN4 after entrancing TS4 with the energy barrier of 11.892 kcal/mol and imaginary frequency of 152i cm^−1^. The elongation of the O_3_–S bond of TS4 almost 0.379 corresponds to a relative increase of 22% in TS4 compared to the IN3 molecule. Obtained specie is known as a complex between singlet molecular oxygen and sulfur dioxide. Then the product complex IN4 can directly transform into the product ^1^O_2_+SO_2_ through O–O bond rupture without any transition state. Singlet molecular oxygen due to spin multiplicity changes and transforms to triplet oxygen through a relaxation process. The reaction thermodynamic parameters indicate that P2 complex, ^1^O_2_  (^1^Δ_g_) + SO_2_, formation is exothermic with enthalpy of reaction, −170.708 kcal/mol, in comparison with the original reactants. In all steps of path P2, the formation of products from reactants is confirmed using related IRC curves.

### 3.5. Formation Pathways of P3 (SO_**4**_ (C_**2v**_))

For production of P3, there is one possible pathway as follows:

 Path 3: R → IN1 → TS1 → IN2 → TS3 → IN3 → TS4 → IN4 → TS5 → P3.

In this path, the formation of the intermediate IN4 is similar to path P2. Then, the IN4 exchange to final product through TS5 by formation of S–O_4_ and O_3_–S bonds. For this step, barrier height is 33.603 kcal/mol. The optimized structure of transition state TS5 shows that O_3_–S bond length is 45% longer than corresponding bond in SO_4_. On the other hand, newly formed bond, O_4_–S, is 51% greater than the corresponding bond in IN4 molecule. Imaginary frequency of TS5 is 455i cm^−1^.

The reaction thermodynamic parameters indicate that P3 formation process is exothermic by 188.042 kcal/mol releasing heat and spontaneous with −179.147 kcal/mol in Gibbs free energy at atmospheric pressure and 298.15 K temperature. The formation of products along the path is confirmed using AIM results and IRC curve.

### 3.6. Formation Pathways of P4 (SO_**4**_ (C_**3v**_))

For production of P4, there is one possible pathway as follows:

 Path 4: R → IN1 → TS1 → IN2 → TS3 → IN3 → TS4 → IN4 → TS5 → P3 → TS6 → P4.

In this path, the formation of the P3 is similar to path P3. Then P3 can be converted into the P4 by angle-variation through TS6 transition state. The angle of O_3_SO_4_ in P3 is 59.6° while it is 106.0° in P4. The breaking bond of O_3_–O_4_ is 1.576 in P3 that extend to 2.176 in TS6. The energy height of this process is 18.231 kcal/mol and imaginary frequency of TS6 is 281i cm^−1^.

The reaction thermodynamic parameters show that P4 formation process is exothermic by 164.857 kcal/mol releasing heat and spontaneous with −156.922 kcal/mol in Gibbs free energy. The SO_4_ (C_2v_) molecule has been confirmed in both the experimental and theoretical studies, while SO_4_ (C_3v_) molecule has been only confirmed in the theoretical studies. In this work, we are obtained both of them that molecule with C_2v_ symmetry is more stable than another. In summary, the ^1^S+^1^O_4_ reaction is energetically feasible to formation of all four products all over corresponding paths which all step of reaction confirm using IRC and AIM calculations.

### 3.7. Topological Analysis of Electronic Density of the Products

AIM topological analysis has been shown to provide important information about many different chemical systems by an analysis of molecular electron density distribution. The electron density, *ρ*(*r*), density Laplacian, ∇^2^ *ρ*(*r*), and bond ellipticity, *ε* = (*λ*_1_/*λ*_2_) − 1, at bond critical points (BCP) have been extracted using AIM topological analysis. The *λ*_1_, *λ*_2_, and *λ*_3_ are the eigenvalues of the Hessian matrix. The first two eigenvalues correspond to the perpendicular curvatures and the latter provides curvatures along the internuclear axis. According to the AIM topological analysis, electron density, *ρ*(*r*), and Laplacian of the electron density, ∇^2^ *ρ*(*r*), are used to describe the strength and the characteristic of the bond, respectively. According to the theory of AIM, the positive value of the Laplacian (∇^2^ *ρ*(*r*)) indicates a weak interaction or an ionic bond; however, the negative value of the Laplacian shows a strong covalent bond between the atoms. The ellipticity shows that the ratio of the rate of density decrease in two directions perpendicular to the bond path at the bond critical point.

The topological analysis of the electronic density were performed for all bonds in the OOSOO, SO_2_ and SO_4_ (C_2v_), and SO_4_ (C_3v_) species as stable products of our studied reaction. The topological characteristics in the BCP's of S–O and O–O bonds are tabulated in Table 4. In addition, the ∇^2^ *ρ*(*r*) values in Table 4 indicate that the every bond in OOSOO has covalent character (∇^2^ *ρ*(*r*) < 0). Their electron density values show that the bond strength manner O–O > S–O is corresponding to the electronic charge densities 0.3606 and 0.2283 au, respectively.

In SO_4_ (C_2v_) molecule, the S–O bond has covalent character with Laplacian of −0.0738 au, whereas the S=O bond has electrostatic interaction nature with Laplacian of the electron density value 0.9675 and O–O bond with 0.3346 au in ∇^2^ *ρ*(*r*) has van der Waals origin. The S=O bond in SO_4_ (C_2v_), in comparison with corresponding bonds in SO_2_ and SO_4_ (C_3v_) molecules, has high electronic density. The results have confirmed that mentioned bond in SO_4_ (C_2v_) is stronger than the corresponding bonds in other species. The Laplacian of S=O bond in SO_2_ and SO_4_ (C_2v_) is very similar and its absolute value is greater than Laplacian of corresponding bond in SO_4_ (C_3v_). The results suggested that S=O bonds in three mentioned molecules have electrostatic nature and can be ionic origin.

The values for the ellipticity of various bonds for three products are listed in Table 4. The high ellipticity (*ε*) for S–O bond in OOSOO molecule shows nonsymmetrical distribution of density about the bond path, whereas low value belongs to the O–O bond which is corresponding with symmetrical distribution of density between two atoms. In SO_4_ (C_3v_) molecule, all of the bonds have the same ellipticity (0.0462 au). This manner shows that bonds have the same symmetry in distribution of *π* bonding character. Comparison of the S–O bond ellipticities in all of species shows that the S–O electron density in OOSOO builds up in a particular orientation and it reveals nonsymmetrical distribution of density between two atoms.

### 3.8. Molecular Orbital Analyses of the Products

The features of the HOMO and LUMO for products are calculated by B3LYP/6-311+G (3df) level in gaseous phase. The HOMO energy characterizes the ability of electron to donate, and the LUMO energy characterizes the ability of electron to accept [36]. The energy gap between HOMO and LUMO characterizes whether the molecules are chemically stable or not. The energy band gap (Δ*ε*) (transition from HOMO to LUMO), ionization potential, and electron affinity of the OOSOO, SO_2_, SO_4_ (C_2v_), and SO_4_ (C_3v_) species can be seen in Table 5. The HOMO-LUMO energy gap results predict that the SO_2_ (0.2073 au) and SO_4_ (C_2v_) (0.2027 au) with large energy gaps (Δ*ε*) are more stable than the OOSOO (0.0.610 au) and SO_4_ (C_3v_) (0.0539 au) molecules. The frontier molecular orbitals of four products are depicted in Figure 4. In OOSOO molecule, the LUMO is localized on the bond between oxygen-sulfur atoms. The HOMO is mainly composed on the p-type atomic orbitals of oxygen and sulfur atoms. In SO_2_ molecule, the HOMO orbital is localized on the bond between oxygen–sulfur atoms while the LUMO is composed in the *π* type orbitals of tree atoms. In addition, results show that HOMO orbital is localized on the *π* type orbitals of oxygen atoms in SO_4_ (C_2v_) while the LUMO is composed mainly just in the *π* type orbitals of two oxygen atoms. Finally, in SO_4_ (C_3v_), the HOMO and LUMO orbitals that are completely similar are localized in the p-type atomic orbitals of oxygen atoms.

## 4. Conclusion

In the percent work, the reaction mechanism of sulfur atom with O_4_ molecule is investigated on the singlet PES. The experimental and theoretical studies show that O_4_ has different structures with various points group. In the neutral structure of tetraoxygen with D_2h_ point group and triplet state are more stable than singlet state and other structures. In spite of numerous attempts, no transition state has been found for the S+O_4_ reaction on the triplet PES. Therefore, the details of theoretical investigation on the singlet PES of S+O_4_ reaction have been carried out at the B3LYP/6-311+G (3df) and CCSD levels. Therefore, in the first step, the sulfur atom on the triplet state, ^3^S+^1^O_4_, is excited to the singlet state, ^1^S+^1^O_4_, and the reaction continues in the singlet state. One stable reactant complex, IN1 (O_2_–S–O_2_), has been considered between the reactants on the singlet PES. Through variety of IN1 transformations, four kinds of products OOSOO, SO_2_+^1^O_2_ (^1^Δ_g_), SO_4_ (C_2v_), and SO_4_ (C_3v_) are obtained which have enough thermodynamic stability. In conclusion, SO_4_ (C_2v_) is the most stable product and its production is spontaneous and exothermic reaction with −188.042 and −179.147 kcal/mol in Gibbs free energy and enthalpy of reaction at the B3LYP level, respectively. In kinetic viewpoint, it is expected that the formation channel of P1 with two transition states and low level energy barriers is favorable process.

## Supplementary Material

Cartesian coordinate of the reactants, products, intermediates and transition states involved in the S+O4 reaction at the B3LYP/6-311+G(3df) level of theory.

## Figures and Tables

**Table 1:** The total energies (in Hartree) and relative energies (in parentheses and kcal/mol) of the reactants, products, and intermediates in the ^1^S+^1^O_4_ reaction on the singlet PES.

Species	B3LYP	CCSD
^ 1^O_4_	−300.6780	−300.1169
^ 1^S+^1^O_4_	−698.7512 (0.000)	−697.7038 (0.000)
^ 3^S+^1^O_4_	−698.8125 (−38.466)	−697.7576 (−33.760)
IN1	−698.9258 (−109.524)	−697.8998 (−122.962)
IN2	−698.8887 (−86.279)	−697.8493 (−91.305)
IN3	−698.9700 (−137.288)	−697.9428 (−149.967)
IN4	−699.0211 (−169.363)	−698.0045 (−188.706)
TS1	−698.8693 (−74.095)	−697.8344 (−81.980)
TS2	−698.8327 (−51.124)	−697.7908 (−54.616)
TS3	−698.8711 (−75.231)	−697.8172 (−71.159)
TS4	−698.9510 (−125.396)	−697.9046 (−125.983)
TS5	−698.9676 (−135.760)	−697.9385 (−147.258)
TS6	−699.0077 (−160.916)	−697.9687 (−166.210)
OOSOO	−698.8598 (−68.119)	−697.7884 (−53.090)
SO_2_+O_2_ (^1^Δ_g_)	−699.0234 (−170.792)	−698.0023 (−187.280)
SO_4_ (C_2v_)	−699.0489 (−186.769)	−698.0316 (−205.672)
SO_4_ (C_3v_)	−699.0127 (−164.089)	−697.9665 (−164.835)

**Table 2:** Topological analyses of O_4_ molecules on the B3LYP level; values are in atomic units.

Species	Bond critical point	*ρ*(*r*)	*λ*_1_	*λ*_2_	*λ*_3_	∇^2^ *ρ*(*r*)	Nature of bond
^ 1^O_4_	Point 1	0.0817	−0.1465	−0.1199	0.6143	0.3479	van der Waals
	Point 2	0.5523	−1.4969	−1.4626	2.2397	−0.7198	Covalent

^ 3^O_4_	Point 1	0.0527	−0.0813	−0.0736	0.3691	0.2142	van der Waals
	Point 2	0.5507	−1.4961	−1.4713	2.2321	−0.7601	Covalent

**Table 3:** The vibrational frequencies (cm^−1^) of the reactants, products, and transition states calculated at the B3LYP level. The value in parentheses refers to experimental data [35].

Species	Frequencies (B3LYP)
^ 1^O_4_	198, 519, 547, 810, 1432, 1624
^ 3^O_4_	248, 279, 398, 626, 1471, 1637
IN1	246, 332, 414, 521, 567, 759, 776, 984, 1041
IN2	156, 184, 370, 546, 554, 666,791, 842, 1010
IN3	73, 254, 411, 602, 652, 775, 921, 930, 1301
IN4	17, 47, 52, 57, 121, 521, 1177, 1371, 1633
TS1	−378, 202, 242, 410, 546, 608, 696, 929, 1042
TS2	−163, 93, 174, 251, 369, 696, 759, 921, 1012
TS3	−591, 93, 369, 399, 520, 608, 767, 970, 1052
TS4	−152, 265, 355, 420, 576, 795, 874, 992, 1233
TS5	−455, 188, 290, 424, 488, 690, 906, 1250, 1442
TS6	−281, 304, 334, 384, 479, 979,986, 1110, 1130
OOSOO	74, 80, 235, 317, 343, 694, 706, 925, 1005
SO_2_	519 (518), 1178 (1151), 1377 (1362)
O_2_ (^1^Δ_g_)	1634 (1484)
SO_4_ (C_2v_)	310, 478 (490), 485 (498), 497, 678 (611), 737 (777), 967 (927), 1279 (1267), 1447 (1437)
SO_4_ (C_3v_)	243, 244, 298, 308, 477, 994, 1035, 1088, 1089

**Table 4:** Topological analyses of products in atomic units.

	OOSOO		SO_2_	SO_4_ (C_2v_)			SO_4_ (C_3v_)
	O–O	S–O	S–O	S=O	S–O	O–O	S=O
*λ*_1_	−0.8486	−0.4070	−0.6145	−0.6264	−0.4161	−0.3970	−0.6069
*λ*_2_	−0.8424	−0.2703	−0.5457	−0.5500	−0.3708	−0.2838	−0.5801
*λ*_3_	1.6457	0.5719	2.1282	2.0939	0.7131	1.0154	1.6849
*ρ*(*r*)	0.3606	0.2283	0.3080	0.3283	0.2373	0.2038	0.3015
∇^2^ *ρ*(*r*)	−0.0453	−0.1054	0.9680	0.9175	−0.0738	0.3346	0.4979
*ε*	0.0073	0.5057	0.1261	0.1389	0.1222	0.3989	0.0462

**Table 5:** HOMO and LUMO energies (in atomic unit) of OOSOO, SO_2_, SO_4_  (C_2v_), and SO_4_  (C_3v_) molecules.

	OOSOO	SO_2_	SO_4_ (C_2v_)	SO_4_ (C_3v_)
*E*_HOMO_	−0.2763	−0.3472	−0.3640	−0.3772
*E*_LUMO_	−0.2153	−0.1399	−0.1613	−0.3233
Δ*ε*	−0.0610	−0.2073	−0.2027	−0.0539

**Table 6:** The thermodynamic data of the S+O_4_ reaction on the singlet PES at the B3LYP method (kcal/mol).

Reaction	Δ*E* ^0^	Δ*H* ^0^	Δ*G* ^0^	*T*Δ*S* ^0^
^ 1^S+^1^O_4_ → OOSOO	−67.694	−68.286	−62.177	−6.108
^ 1^S+^1^O_4_ → SO_2_+^1^O_2_ (^1^Δ_g_)	−170.708	−170.708	−173.338	2.629
^ 1^S+^1^O_4_ → SO_4_ (C_2v_)	−187.450	−188.042	−179.147	−8.896
^ 1^S+^1^O_4_ → SO_4_ (C_3v_)	−164.265	−164.857	−156.922	−7.935

## References

[1] Lewis GN (1924). The magnetism of oxygen and the molecule O_4_. *Journal of the American Chemical Society*.

[2] Varandas AJC, Llanio-Trujillo JL (2002). On triplet tetraoxygen: ab initio study along minimum energy path and global modelling. *Chemical Physics Letters*.

[3] Prasad O, Sinha L, Misra N (2009). Study of electrostatic potential surface and molecular orbitals of O_4_ nano cluster by first principles. *Scholars Research Library*.

[4] Long CA, Ewing GE (1973). Spectroscopic investigation of van der waals molecules. I. The infrared and visible spectra of (O_2_)_2_. *The Journal of Chemical Physics*.

[5] Peterka DS (1999). Unraveling the mysteries of metastable O_4_. *Journal of Chemical Physics*.

[6] Dunn KM, Scuseria GE, Schaefer HF (1990). The infrared spectrum of cyclotetraoxygen, O_4_: a theoretical investigation employing the single and double excitation coupled cluster method. *The Journal of Chemical Physics*.

[7] Hernández-Lamoneda R, Ramírez-Solís A (2000). Reactivity and electronic states of O_4_ along minimum energy paths. *Journal of Chemical Physics*.

[8] Pillay D, Wang Y, Hwang GS (2006). Prediction of tetraoxygen formation on rutile TiO_2_(110). *Journal of the American Chemical Society*.

[9] Seidl ET, Schaefer HF (1992). Is there a transition state for the unimolecular dissociation of cyclotetraoxygen (O_4_)?. *The Journal of Chemical Physics*.

[10] Aquilanti V, Ascenzi D, Bartolomei M (1999). Molecular beam scattering of aligned oxygen molecules. The nature of the bond in the O_2_—O_2_ dimer. *Journal of the American Chemical Society*.

[11] Arnold SJ, Ogryzlo EA, Witzke H (1964). Some new emission bands of molecular oxygen. *The Journal of Chemical Physics*.

[12] Helm H, Walter CW (1993). Observation of electronically excited states of tetraoxygen. *The Journal of Chemical Physics*.

[13] Cacace F, de Petris G, Troiani A (2001). Experimental detection of tetraoxygen. *Angewandte Chemie International Edition*.

[14] Bevsek HM, Ahmed M, Peterka DS, Sailes FC, Suits AG (1997). Direct detection and spectroscopy of O_4_. *Faraday Discussions*.

[15] Adamantides V, Neisius D, Verhaegen G (1980). Ab initio study of the O_4_ molecule. *Chemical Physics*.

[16] Røeggen I, Nilssen EW (1989). Prediction of a metastable D_3h_ form of tetra oxygen. *Chemical Physics Letters*.

[17] Gorelli FA, Ulivi L, Santoro M, Bini R (1999). The *ε* phase of solid oxygen: evidence of an O_4_ molecule lattice. *Physical Review Letters*.

[18] Lundegaard LF, Weck G, McMahon MI, Desgreniers S, Loubeyre P (2006). Observation of an O_8_ molecular lattice in the *ε* phase of solid oxygen. *Nature*.

[19] Hotokka M, Pyykkö P (1989). An ab initio study of bonding trends in the series BO_3_
^3-^, CO_3_
^2-^, NO_3_
^−^ and O_4_(D_3h_). *Chemical Physics Letters*.

[20] Jubert AH, Varetti EL (1986). On the possible existence of the O_4_ molecule with D_3h_ symmetry. *Anales de Química*.

[21] Pruppacher HR, Klett JD (1980). *Microphysics of Clouds and Precipitation*.

[22] Wong MW, Steudel Y, Steudel R (2007). Structures and vibrational spectra of the sulfur-rich oxides S_*n*_O (*n* = 4–9): the importance of *π**-*π** interactions. *Chemistry A*.

[23] Jacob A, Winkler CA (1972). Kinetics of the reactions of oxygen atoms and nitrogen atoms with sulphur trioxide. *Journal of the Chemical Society, Faraday Transactions*.

[24] McKee ML (1993). Computational studies on SO_4_ and S_2_O_3_. *Journal of the American Chemical Society*.

[25] Schriver L, Carrere D, Schriver A, Jaeger K (1991). Matrix-isolation photolysis of SO_2_, O_3_ and H_2_O: evidence for the H_2_O:SO_3_ complex. *Chemical Physics Letters*.

[26] Kugel R, Taube H (1975). Infrared spectrum and structure of matrix-isolated sulfur tetroxide. *Journal of Physical Chemistry*.

[27] Levitt LS (1953). A new mechanism for persulphate oxidations. *Canadian Journal of Chemistry*.

[28] Frisch MJ, Trucks GW, Schlegel HB (2003). *Pople JA Gaussian 03, Revision B.03*.

[29] Becke AD (1993). A new mixing of hartree-fock and local density-functional theories. *The Journal of Chemical Physics*.

[30] Scuseria GE, Schaefer HF (1989). Is Coupled Cluster Singles and Doubles (CCSD) more computationally intensive than Quadratic Configuration Interaction (QCISD)?. *The Journal of Chemical Physics*.

[31] Scuseria GE, Janssen CL, Schaefer HF (1988). An efficient reformulation of the closed-shell Coupled Cluster Single and Double Excitation (CCSD) equations. *The Journal of Chemical Physics*.

[32] Gonzalez C, Schlegel HB (1990). Reaction path following in mass-weighted internal coordinates. *Journal of Physical Chemistry*.

[33] Parr RG, Yang W (1989). *Density-Functional Theory of Atoms and Molecules*.

[34] Biegler-Konig F, Schoenbohm J (2002). *AIM2000*.

[35] Johnson RD, NIST Computational chemistry comparison and benchmark database. http://cccbdb.nist.gov/.

[36] Atkins PW (2001). *Physical Chemistry*.

