# Supplementary material for: Prediction of Tetraoxygen Reaction Mechanism with Sulfur Atom on the Singlet Potential Energy Surface
Source: ScientificWorldJournal. 2014 Jan 23;2014:912391. doi: 10.1155/2014/912391 (PMC3920618; doi:10.1155/2014/912391)
Supplement: Supplementary file 1 — Cartesian coordinate of the reactants, products, intermediates and transition states involved in the S+O4 reaction at the B3LYP/6-311+G(3df) level of theory. [file 912391.f1.docx]

**Supporting Information of**

**Prediction of tetraoxygen reaction mechanism with sulfur atom on the singlet potential energy surface**

Ashraf Khademzadeh, Morteza Vahedpour,^*^ Fereshte Karami

Department of Chemistry, University of Zanjan, Zanjan, Iran

^*^ To whom correspondence should be addressed. Tel.: +98 241 5152631; fax: +98 241 5152477. E-mail adresses: [vahed@znu.ac.ir](mailto:vahed@znu.ac.ir) (Morteza Vahedpour)

Cartesian coordinate of the reactants, products, intermediates and transition states involved in the S+O_4_ reaction at the B3LYP/6-311+G(3df) level of theory.

^3^O_4_

O 0.00000000 0.60070600 1.06057600

O 0.00000000 0.60070600 -1.06057600

O 0.00000000 -0.60070600 1.06057600

O 0.00000000 -0.60070600 -1.06057600

^1^O_4_

O 0.00000000 0.96694800 0.60035300

O 0.00000000 -0.96694800 0.60035300

O 0.00000000 0.96694800 -0.60035300

O 0.00000000 -0.96694800 -0.60035300

IN1

O 0.25146600 -0.30470900 -1.31854800

O 0.11923300 -0.49227600 1.26944100

O 1.36928700 0.29390100 0.88774800

O -1.21007100 -0.42732600 -0.89981600

S -0.08779000 0.45833400 0.02360500

IN2

O 0.05170100 0.26588900 -1.42737600

O 0.39620700 0.27710500 1.16366900

O 1.49227500 -0.56144100 1.04221200

O -0.77096500 -0.89212000 -0.86145000

S -0.62414900 0.45464900 0.04203600

IN3

O 1.46661600 0.63197000 0.15061700

O -1.60521500 -0.21893400 0.55360800

O 0.27882000 1.17802400 -0.48837000

O 0.90494000 -0.69927200 0.32267600

S -0.56641100 -0.29664300 -0.42911500

IN4

O 0.07662600 -0.15451800 0.12444800

O -0.09887500 -0.01509300 3.60483400

O 1.01848900 0.19645900 -0.53325900

O -1.62934100 1.64121500 2.58165400

S -0.28990800 1.20261100 2.86487700

TS1

O 0.00958900 0.07431000 -0.01784900

O -0.05797200 0.03959900 2.53802400

O 1.42295400 -0.06672700 2.19221900

O -1.33529500 -0.58773100 0.26482400

S -0.73621300 0.56282700 1.30142600

TS2

O 0.00257800 0.02082900 -0.00578200

O 0.00181400 0.01613000 4.71665200

O 0.59125200 -0.00212900 3.52345300

O 0.55339800 0.62682200 1.12958500

S -0.42862300 0.21945700 2.28706400

TS3

O 1.74365900 0.57831100 0.08110400

O -1.78492200 0.23334300 0.24299100

O -0.28789900 1.00191600 -0.19649700

O 1.22693800 -0.64164100 0.11071800

S -0.42531900 -0.56957400 -0.12936000

TS4

O 0.00133500 -0.00965900 0.00096900

O -0.00571000 -0.01318100 3.53896100

O 1.30426800 -0.00209100 0.35059700

O -0.60563200 -0.34796500 1.23751000

S 0.67384900 -0.34592800 2.30315600

TS5

O -1.86569400 -2.00735600 -2.66989300

O -0.59429000 -1.01428900 -0.67413600

O -0.44556500 -2.15792400 -3.13877400

O -2.52223900 -2.58223400 -0.38608900

S -1.64087300 -1.81396900 -1.19406200

TS6

O -1.67813400 -1.53674800 0.33531100

O -3.99128100 -1.31539100 -0.20182400

O -2.63546400 0.66401000 -0.37594000

O -2.05404900 -1.17382800 -1.78075300

S -2.67190200 -0.78197400 -0.48125200

P1 (OOSOO)

O 0.08030300 0.17463100 -0.08299800

O 0.05462900 0.16761300 4.73383800

O 0.57661300 -0.08288200 3.51959800

O 0.51367100 0.54366000 1.13588900

S -0.48290400 0.10616400 2.32228400

P3 (SO_4_ (C_2v_))

O -0.52702300 0.32609300 -1.24975000

O 0.74097800 1.08647900 0.80316400

O 0.70979500 -0.63131700 -1.05388000

O -0.79112000 -0.85816800 0.97012000

S 0.01357700 0.02566300 0.21066900

P4 (SO_4_ (C_3v_))

O 0.02049400 -0.00304900 0.00886700

O 0.00579700 0.01991600 2.35528300

O 2.12726600 -0.00289400 1.35344800

O 0.86378600 1.94449300 1.01093500

S 0.75416800 0.48980100 1.18194300
